# Salivary Hormones and Quality of Life in Female Postmenopausal Burning Mouth Patients—A Pilot Case-Control Study

**DOI:** 10.3390/dj8040111

**Published:** 2020-10-01

**Authors:** Božana Lončar-Brzak, Valentina Vidranski, Ana Andabak-Rogulj, Danica Vidović-Juras, Ivana Todorić-Laidlaw, Dragana Gabrić, Ivana Škrinjar

**Affiliations:** 1 Department of Oral Medicine, School of Dental Medicine, University of Zagreb, 10000 Zagreb, Croatia; bozana.loncar@gmail.com (B.L.-B.); dvjuras@gmail.com (D.V.-J.); 2 Department of Nuclear Medicine and Oncology, University Clinical Hospital Sisters of Mercy, 10000 Zagreb, Croatia; vvidranski@gmail.com; 3 Department of Oral Medicine, University Clinical Hospital Zagreb, 10000 Zagreb, Croatia; skrinjar.ivana@gmail.com; 4 Department of Psychotic Disorders (for Women), University Psychiatric Hospital Vrapce, 10000 Zagreb, Croatia; itodoriclaidlaw@gmail.com; 5 Department of Oral Surgery, School of Dental Medicine, University of Zagreb, 10000 Zagreb, Croatia; dgabric@sfzg.hr

**Keywords:** burning mouth syndrome, salivary hormones, quality of life

## Abstract

The objective of our study was to investigate salivary levels of estradiol, progesterone and dehydroepiandrosterone (DHEA), and quality of life, in female postmenopausal women with burning mouth syndrome. The study included new patients diagnosed with burning mouth syndrome and excluded local and systemic causes. Unstimulated saliva samples were taken in the morning from 9 AM and 11 AM and immediately frozen for hormone analysis. The patients filled out a self-perceived quality of life questionnaire Oral Health Impact Profile-14 and determined the intensity of mucosal symptoms according to the visual-analog scale grading 0 to 10. A total of 40 patients were included. The study group had significantly lower levels of salivary estradiol. No difference was observed in levels of progesterone and DHEA between the groups. The levels of salivary hormones did not exhibit a significant correlation according to the Spearman correlation test with a self-perceived quality of life questionnaire (OHIP-14) in the study group or in the control group. Further research on a larger number of patients is needed to verify these results. This information might help to enable more precise and efficient treatment.

## 1. Introduction

Burning mouth syndrome (BMS) is an unpleasant condition described as painful or burning sensations of clinically unchanged oral mucosa. The intensity of symptoms oscillates throughout the day [1,2], and they are often accompanied by mouth dryness and taste disturbance [3,4]. It is necessary to exclude local and systemic potential causes to establish the diagnosis [1,2]. The literature offers non-unique criteria for diagnosis without a definitive test, so establishing the diagnosis and determining treatment is challenging for clinicians.

So far, BMS has been considered an idiopathic disorder. Various results from the literature point to a complex correlation with the psychological profiles of the patients, stressful events, and local and systemic causes [2,3,4].

Up to 15% of adults are affected by this disorder [5,6]. Females, especially perimenopausal and postmenopausal women, are predominantly affected, which initiated research of female sex hormones and BMS [7]. A literature search has revealed several studies that have explored the complex connection between psychological factors, hormonal status and BMS, but the results are limited and inconclusive.

A possible explanation for the neuroendocrine etiology of BMS lies in the hypothalamic–pituitary–adrenal (HPA) axis, which regulates responses to neuroendocrine stress [8] and its crosstalk to the hypothalamic–pituitary–gonadal (HPG) axis. Long-term dysregulation of this system impairs the psychological and physical health of the individual [9,10]. Bale and Epperson [11] have shown that mechanisms involved in stress responses differ between the sexes. Results in the literature regarding HPA and HPG axis responses in BMS patients are limited. A few studies have shown altered levels of female gonadal hormones in the serum and saliva of BMS patients, but with unequivocal results [12,13,14,15].

Estradiol, progesterone and dehydroepiandrosterone (DHEA) are circulating steroid hormones that are involved in multiple processes in the body. Estradiol participates in the regulation of cognitive and socioemotional processes as well as psychopathology [16], while progesterone is involved in brain development and behaviour [17]. Estradiol and progesterone, among other functions, mutually participate in the regulation of pain perception [18], but it seems that the pituitary independently regulates their levels through a complex HPG axis [19,20].

Dehydroepiandrosterone (DHEA) is a precursor of powerful androgens and estrogens. It regulates the amount of adipose tissue in the body and immune response and modulates sensory and psychologic processes [21,22,23]. The research has shown that DHEA levels are decreased in some psychologic disorders such as depression, schizophrenia, Alzheimer’s disease and others [24].

The aim of our study was to investigate salivary levels of estradiol, progesterone and dehydroepiandrosterone; the intensity of symptoms; and self-perceived quality of life in female postmenopausal women with burning mouth syndrome and in an age- and sex-matched control group without BMS. We hypothesised that patients with BMS will have lower levels of estradiol, progesterone and dehydroepiandrosterone than the control group and lower quality of life.

## 2. Materials and Methods

This study was approved by the ethical committee of the University Hospital Center Zagreb (No. 17/20, 10/11/2019). Our research was registered at the U.S. National Institutes of Health (clinicaltrials.gov) (trial identifier: NCT04535973). Each participant signed informed consent according to the Declaration of Helsinki.

This was a case-control pilot study that included a total of 40 patients: 28 female postmenopausal patients newly diagnosed with burning mouth syndrome and excluded local and systemic causes [2], and control group of 12 postmenopausal women without burning symptoms. Only female postmenopausal patients were included in the study, in order to have more homogeneous groups and to exclude hormone variations during different stages of menstrual cycle. Excluding criteria were patients taking anxiolytics, anticonvulsants or antidepressants, or undergoing hormonal therapy that could affect hormone levels.

Participants were given oral instructions on how to collect adequate saliva samples according to standardised manufacturer protocol for saliva collection, storage and analysis.

Patients fasted from food, cigarettes and chewing gum for at least one hour and from alcohol for a minimum of 12 h and 10 min before sampling patients also had to rinse their mouth with water.

Saliva was collected between 9 AM and 11 AM in order to minimise diurnal variability, by unstimulated passive drool (gold standard), tilting the head forward and allowing the saliva to pool on the floor of the mouth. Saliva samples were collected in 15 mL TPP^®^ conical graduated, sterile tubes (TPP Techno Plastic Products AG, Trasadingen, Switzerland) with no additives. After collection, we refrigerated the samples within 30 min and froze them only once at −20 °C within 4 h of collection.

On the day of assay, we thawed the saliva samples completely at room temperature (20–23 °C) and vortexed and centrifuged them for 10 min at 1500× *g* at 4 °C on Hettich ROTINA35 centrifuge (Hettich, Germany).

Salivary analysis was performed using competitive ELISA (enzyme-linked immunosorbent assay) immunoassay kit for the quantitative measurement of Progesterone (kit 1–1502), 17β-Estradiol (kit 1–3702) and DHEA (kit 1–1202), Salimetrics Assay (Salimetrics, LLC, State College, PA, USA). Quality controls were ran with each assay.

Progesterone has analytical sensitivity of assay 5 pg/mL, assay range up to 2430 pg/mL, precision CV = 8.4%, 17β-Estradiol analytical sensitivity 0.1 pg/mL, assay range up to 32 pg/mL, precision CV = 7.0% and DHEA analytical sensitivity 5 pg/mL, assay range up to 1000 pg/mL and precision CV = 5.8%.

Plate was read at 450 nm on plate reader VIKTOR2 Wallac 1420 Multilabel Counter (Perkin Elmer, MA, USA). The amount of detected Progesterone, 17 β-Estradiol and DHEA Enzyme Conjugate is inversely proportional to the amount of hormones present in the sample.

The participants filled out a self-perceived quality of life questionnaire Oral Health Impact Profile-14 and determined the intensity of mucosal symptoms according to the visual-analog scale grading 0 to 10 (0—without symptoms, 10—the worst possible symptoms).

Statistical analysis was performed by the MedCalc statistical software version 18.10.2 (MedCalc Software, Ostend, Belgium). The level of significance was set at 5%, where *p* values of 0.05 were considered to be significant. Correlation between hormone level and self-perceived quality of life questionnaire (OHIP-14) was calculated by the Spearman correlation test.

## 3. Results

Median age of BMS patients was 64 (51–75) years. Median age of controls was 63 (53–72) years. There was no difference regarding age and sex between groups. Difference between salivary 17 β-estradiol levels between study group and controls was significant (Table 1). There were no differences in salivary levels of progesterone (Table 2) and DHEA (Table 3) between the groups.

Median value of symptoms intensity on VAS scale was 6 (1–10). The levels of salivary hormones did not exhibit a significant correlation according to the Spearman correlation test with self-perceived quality of life questionnaire (OHIP-14) (*p* < 0.05, Table 4).

## 4. Discussion

In this study, we examined the level of salivary HPA-axis hormones and quality of life in postmenopausal women. Our results have shown that postmenopausal women with BMS have significantly lower salivary estradiol levels than postmenopausal women in control group (*p* < 0.05). This is in concordance with the results of Gao et al. [14], who reported lower estradiol blood levels in postmenopausal patients with BMS than in the control. Nasri C et al. [25] and Lee and Chon [15] found that women with burning mouth complaints have low estradiol levels and self-reported sleeping disorders. The level of estradiol decreases in menopause and affects psychological condition and the perception of pain. Yoshida et al. [26] showed that lower estradiol levels in serum of BMS postmenopausal women correlated with depression, stress and decrease in salivary flow. Because of this, it is suggested that decrease of estradiol in menopause could be related to BMS [15]. Our results support this finding, since we determined significantly lower estradiol in BMS group. Further research should clarify the causal relationship of BMS and estradiol (i.e., which one is the cause and which one is the effect).

Unlike these results, Kim et al. [12] did not find significant difference in estradiol levels in samples of unstimulated saliva between study and control groups. They found significant difference only in samples of stimulated saliva. Our reagent instructions stated that estradiol levels should be measured in unstimulated saliva, so we cannot compare our results with these ones.

We did not observe significant difference between our groups regarding the status of local neuroactive steroids progesterone and DHEA. There are only a few studies investigating progesterone level in BMS patients in the literature, but some of them did not have a control group, so we cannot compare our results with them [27]. Regarding progesterone levels, our results are consistent with those of Kim et al. [12], who also did not find significant difference in progesterone levels between female BMS patients and control group.

Our results did not show a significant difference in salivary DHEA between the groups. Regarding DHEA levels, results from the literature differ. Some studies have found decreased levels of DHEA in BMS patients [13,28], while other have found increased values [12]. Since DHEA modulates sensory and psychologic processes [21,22,23], affects pain perception [29] and has anxiolytic and antidepressive effects [30], it is possible that this is the reason our patients, who did not differ significantly in OHIP-14 values, also did not differ in DHEA levels.

Results from the literature show that most patients with BMS share certain psychological and psychiatric characteristics. Different studies have shown that patients with these difficulties are more prone to stress, depression and catastrophizing [31,32,33,34]. Unlike these, some authors have not shown significant difference among the patients with BMS and control group regarding hopelessness and depression [13].

This is in accordance with our results, which did not show significant difference in self-perceived quality of life between study and control group. We have several possible explanations for this. It is possible that, because the patients included in the study were newly diagnosed, they were unaware of the chronic nature of the disease. In our experience, newly diagnosed patients with BMS often expect to get a definite cure for their symptoms. They believe the symptoms are caused by allergy or some kind of oral infection that can be cured. Therefore, the disorder still did not have a great impact on their quality of life. Additionally, the intensity of their symptoms was modest (median value was 6 on VAS scale) and maybe could have been more easily tolerated by the patients.

Another explanation for this finding is that the levels of DHEA did not significantly differ between the groups. Among its multiple roles in the organism, DHEA has anxiolytic and antidepressive effect [30] that might have influenced patients’ perception of quality of life. Yet, quality of life is multifactorial and cannot be explained only by levels of DHEA. Among other possible reasons, psychological profiles of patients also affect their perception of the disease, but unfortunately we did not evaluate this in this study.

The present study has some limitations. Both groups consisted of a relatively small number of patients. COVID-19 epidemic limited our regular clinical work, and in the meantime the reagents were at risk of expiring so we had to finish the research. Yet, the results are worth reporting because the number of studies in the literature reporting the level of these hormones in BMS patients is limited. Additionally, our results refer to a specific group of patients that are most frequently affected with BMS.

## 5. Conclusions

Different salivary markers are being evaluated as diagnostic or therapeutic targets for BMS. Our results have shown significantly lower salivary estradiol levels in postmenopausal women with BMS. Further research on a larger number of patients is needed to verify these results. This information might help to enable more precise and efficient treatment.

## Figures and Tables

**Table 1:** Salivary 17 β-estradiol levels in burning mouth syndrome (BMS) patients and controls.

	BMS Patients	Controls	*p* *
Salivary 17 β-estradiol (pg/mL) median (range)	0.9 (0.4–2.3)	2.5 (1.8–7.4)	0.0003
N	28	12	

* Mann–Whitney test.

**Table 2:** Salivary progesterone levels in BMS patients and controls.

	BMS Patients	Controls	*p* *
Salivary progesterone (pg/mL) median (range)	42.2 (4.6–170.6)	64 (23.5–121.7)	>0.05
N	28	12	

* Mann–Whitney test.

**Table 3:** Salivary dehydroepiandrosterone (DHEA) levels in BMS patients and controls.

	BMS Patients	Controls	*p* *
Salivary DHEA (pg/mL) median (range)	160.2 (52.1–517.6)	163.3 (119.8–459.1)	>0.05
N	28	12	

* Mann–Whitney test.

**Table 4:** Correlation between self-perceived quality of life questionnaire (OHIP-14) scores and salivary hormones.

		Salivary Hormones		
		17 β-Estradiol	Progesterone	DHEA
OHIP-14 scores	Correlation coefficient *r*	−0.33	0.29	0.02
	Significance level *p* *	0.11	0.15	0.94
	95% Confidence interval for *r*	−0.64 to 0.08	−0.1118 to 0.6191	−0.38 to 0.41

* Spearman correlation.
